# Rheological and Mechanical Characterization of 3D-Printable Solid Propellant Slurry

**DOI:** 10.3390/polym16050576

**Published:** 2024-02-20

**Authors:** Alessandra Zumbo, Leonardo Stumpo, Paola Antonaci, Andrea Ferrero, Filippo Masseni, Giovanni Polizzi, Giacomo Tetti, Dario Pastrone

**Affiliations:** 1 Department of Mechanical and Aerospace Engineering, Politecnico di Torino, Corso Duca degli Abruzzi 24, 10129 Torino, Italy; 2 Department of Structural, Geotechnical and Building Engineering, Politecnico di Torino, Corso Duca degli Abruzzi 24, 10129 Torino, Italy

**Keywords:** composite solid propellant, polybutadiene, photo-polymerization, polymer chemistry, additive manufacturing, rheology

## Abstract

This study delves into the rheological and mechanical properties of a 3D-printable composite solid propellant with 80% wt solids loading. Polybutadiene is used as a binder with ammonium sulfate, which is added as an inert replacement for the ammonium perchlorate oxidizer. Further additives are introduced to allow for UV curing. An in-house illumination system made of four UV-A LEDs (385 nm) is employed to cure the resulting slurry. Rheological and mechanical tests are conducted to evaluate the viscosity, ultimate tensile strength and strain, and compression behavior. Viscosity tests are performed for both pure resin and complete propellant composition. A viscosity reduction factor is obtained for the tested formulations when pre-heating slurry. Uniaxial tensile and compression tests reveal that the mechanical properties are consistent with previous research. Results emphasize the critical role of temperature and solid loading percentage. Pre-heating resin composites may grant a proper viscosity reduction while keeping mechanical properties in the applicability range. Overall, these findings pave the way for the development of a 3D printer prototype for composite solid propellants.

## 1. Introduction

**Figure 1:**
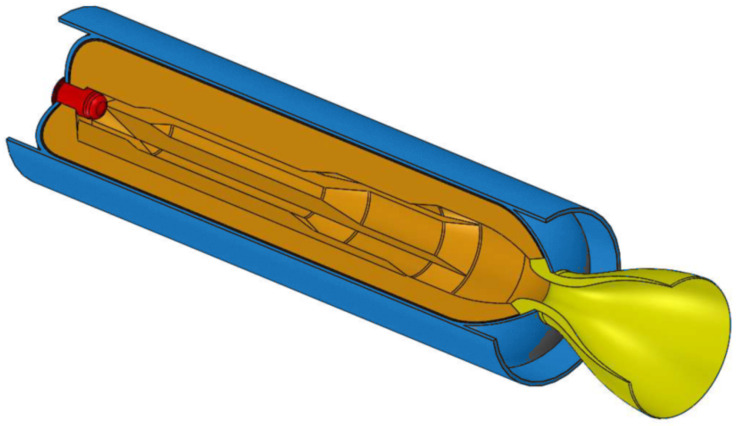
Simplified perspective three-quarter section view of a typical solid propellant rocket motor showing propellant grain (orange), nozzle (yellow), igniter (red) and case with insulation layer (blue) (adapted from Ref. [4]).

In the space propulsion field, a solid rocket motor, as shown in Figure 1, is composed by a casing that encloses the solid propellant along with other components. The case, which serves as a combustion chamber, also comprises an igniter, an insulation layer and a nozzle. The composition and geometrical configuration of the grain impact motor performance parameters [1,2]. The composite propellant grain consists of oxidizing particles combined with fuel that include a polymeric binder and, possibly, metal powder. The traditional method of producing such propellants requires a premix of all the ingredients except for the oxidizer and curing agent. The final mixing with the oxidizer and the curing agent occurs before casting it into the mold or motor case and curing in an oven or furnace [1,2,3]. The typical cast molding process requires the use of harmful chemicals, long processing times and limitations on possible geometries [1]. These intrinsic limits in terms of production flexibility, promptness and grain geometry, together with concerns about toxicity, have prompted researchers to investigate novel solutions.

The implementation of additive manufacturing techniques has yielded significant advancements in a number of sectors in recent years [5,6,7]. Furthermore, solid propellant grains are of particular interest in different field of energetics, where rapid gas production with compact devices is required (e.g., beside space propulsion, air-bags and fire extinguishers). Specifically, recent efforts for propulsion applications have focused on exploring innovative binders and proof of concepts [8,9,10,11]. In parallel, photo-polymerization has lately emerged as a viable alternative to traditional manufacturing in a variety of industries [12,13,14,15,16,17]. Finally, extensive reviews have been performed on the application of various 3D printing technologies in energetic materials [6,18,19].

It is within this context that a patented manufacturing process based on deposition and UV-activated polymerization for solid rocket motors was developed [20]. The initial phase of the project focused on the applicability of UV photopolymerization to create solid grains with suitable mechanical and physical properties [4,21]. Various photocurable resins were considered as binders for highly loaded composite propellants. These binders were mixed with powdered ammonium sulfate in varying amounts to create inert solid propellant. Curing kinetics and mechanical properties were investigated. Although the mechanical characteristics of the composites were found to be lower than those of typical isocyanate-cured compositions, the materials provided in the study offer a starting point for further modification and optimization. After testing the resins, polybutadiene was chosen for the subsequent project phases due to its performance and applicability.

On the basis of the results previously obtained, the present research represents the initial steps toward the ultimate objective of prototyping a suitable deposition system to be integrated with the UV-light illuminator. Upon evaluating various options [22], a top-feed deposition nozzle emerged as the most promising method for material delivery. However, this approach introduces complexities related to moving highly viscous heterogeneous suspensions. As a result, extensive tests should be performed to determine feasible methods to reduce viscosity. In particular, the investigation of the role of temperature in viscosity is crucial to gain insights into the thermal behavior of the propellant, which directly correlates with its printability during the 3D-printing process. Understanding these temperature-dependent viscosity changes is instrumental in optimizing the printing parameters and ensuring the successful production of solid propellant grains. Having achieved a reduction in viscosity through temperature modulation, the next critical step involves examining the mechanical properties of the material when printed at elevated temperatures. This comparative analysis against reference values at ambient temperature for the same composition is indispensable for determining the optimal temperature conditions during the 3D-printing process. The synergy between rheological and mechanical assessments plays a crucial role in advancing the understanding of material behavior, thereby facilitating informed decisions in the development of an efficient 3D printing system for the photocurable solid propellant.

With a primary focus on temperature effects, the current study offers insights on viscosity, ultimate tensile strength and strain, and compression behavior. These findings provide valuable guidance for optimizing the 3D-printing process of photocurable composite propellants, not only addressing the specific challenges within propulsion technology but also contributing meaningfully to the broader field of material science and additive manufacturing.

## 2. Experimental Part

### 2.1. Materials

Every formulation tested in this work consists of polybutadiene binder loaded with 80% wt ammonium sulfate (AS) as inert replacement for ammonium perchlorate (AP). The AS salt was chosen as an inert replacement for solid oxidizer because it had comparable solubility and polymer bond strength, and both AS and AP crystals have been observed to be transparent in UV-visible spectra [23].

Polybutadiene (Mn = 5000) (PB), bis-(2,4,6-trimethylbenzoyl) phenylphosphine oxide (BAPO), pentaerythritol tetrakis (3-mercaptopropionate) (PTTM, simply called thiol in the following) and AS were purchased from Sigma Aldrich (Milan, MI, Italy). The AS to be added to the resin binder PB should have varying particle sizes in well-defined proportions, while they are supplied in crystals with a size exceeding 800 microns. Thus, the purchased AS was further processed into coarse and fine powder (220 μm and 20 μm) by SPLab Politecnico di Milano. The two different particles sizes are needed to optimize filling: the smaller particles are used to fill the gaps left by the larger ones.

For mechanical tests (see Section 2.2.3 and Section 2.2.4), the compound to pour into the molds was obtained by combining the resin (PB) with 80 weight percent of AS powder (80% coarse + 20% fine) and manually stirring to obtain a homogeneous mixture. Then, the addition of thiol allowed the photocuring reaction of thiol-ene to take place, and BAPO (photo-initiator) was finally added. The quantities for thiol and BAPO are given in Parts per Hundred parts Resin (%phr) and are, respectively, 14%phr and 4%phr. A homogeneous mixture was achieved after 5 min of manual stirring. The identical process is used to prepare samples for viscosity tests (see Section 2.2.1) with the exception of the photo-initiator (BAPO), which was purposefully left out to prevent crosslinking during testing.

### 2.2. Methods

#### 2.2.1. Viscosity Measurements

In order to examine the impact of temperature on the composition of the propellant and its consequential influence on the printability of 3D solid propellants, a comprehensive study on viscosity was carried out in three distinct phases. The formulation for the three test cases is shown in Table 1. First, viscosity measurements were performed on pure resin (PB) in order to define a standard for tests duration and vessel diameter Φ (test case #1); secondly, the propellant formulation was characterized for the 80% wt loaded formulation (20PB-80AS, test case #2). Finally, the effect of thiol addition was evaluated, providing a sensible range for the whole formulation’s viscosity (20PB-80AS with thiols, test case #3). For every formulation, viscosity was measured using a digital rotary viscometer SAVISC 152-2, by SAMA tools (Viareggio, LU, Italy), for temperatures ranging from 25 to 90 °C. The test procedure is as follows: after attaching a spindle to the rotational viscometer and selecting a speed, the torque (usually expressed in percentage of maximum torque value, which depends on the speed and rotor chosen) and dynamic viscosity are obtained by the user for a sample posed in a cylindrical vessel. The resulting torque is dependent on the spindle geometry, rotational speed and sample viscosity.

The arbitrary dimensions of the vessel used for testing pose a challenge in accurately measuring the liquid viscosity. According to the ASTM D2196-20 standard [24], a 600 mL beaker should be employed. However, the time-consuming procedure of obtaining correct particle sizes for AS resulted in limited resources available for conducting the tests. Therefore, following earlier research findings, the decision to not conform to the ASTM standard was made. In fact, Refs. [25,26] show that it is possible to measure viscosity in containers with less-than-regulated diameters, just introducing a proper correction coefficient for the accuracy of the results. Thus, a smaller vessel in PPCF material, purchased by Filoalfa (Turin, TO, Italy), internal diameter Φ=3.5 cm) was manufactured using 3D technology and utilized for all the experiments.

**Figure 2:**
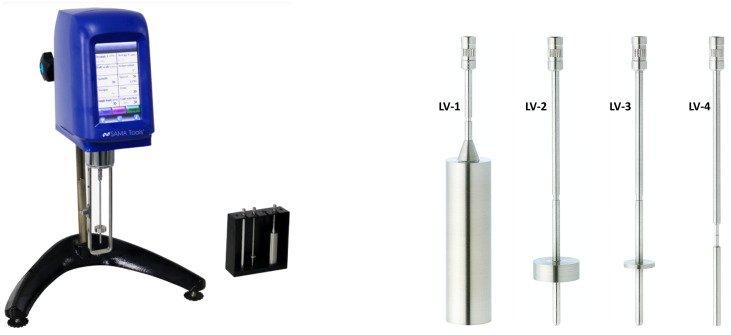
Rotary viscometer SAVISC 152-2 by SAMA tools (Viareggio, LU, Italy) and standard LV spindles.

An overview of the used parameters necessary to fully define a test is given in Table 1, while the viscometer and spindles used are shown in Figure 2.

**Figure 3:**
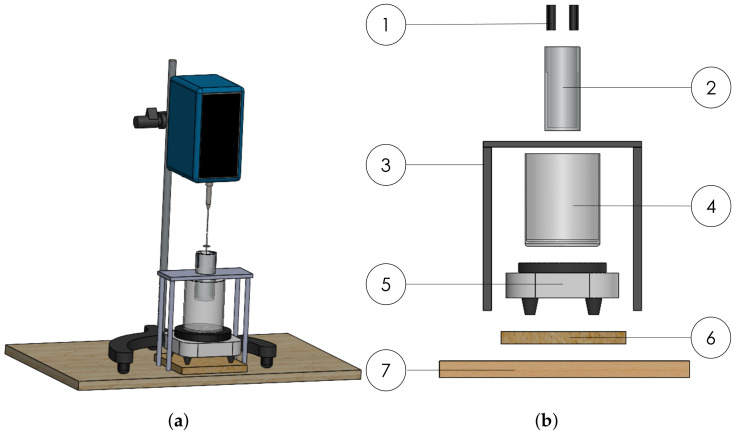
Setup for viscosity analysis. (**a**) Test bench including SAVISC 152-2 rotational viscometer, equipped with spindle nr 3 before immersion into the vessel. (**b**) Exploded front view of test bench: (1) thermometers probe support, (2) PPCF vessel, (3) rectangular support, (4) beaker, (5) heating plate, (6) insulation block, and (7) work bench.

**Figure 4:**
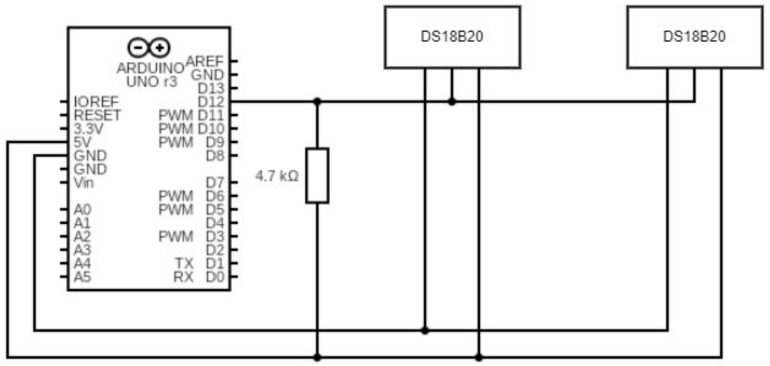
Arduino configuration for the DS18B20 thermometers.

Every viscosity analysis is conducted within a temperature range varying from ambient temperature $T_{ambient}=$ 25 °C to $T_{final}=$ 90 °C through the use of a thermostatic bath. The test bench is illustrated in Figure 3a,b, which highlights in detail the setup for viscosity tests. The two DS18B20 thermometers used for temperature detection are immersed at different depths inside the cylindrical container near the walls, and an Arduino board is used to measure the temperature output, as shown in the diagram in Figure 4. The two thermometers are positioned to avoid interfering with the test sample: guides facilitate ring insertion to maintain proximity between temperature probes and the wall. The water inside a 500 mL beaker, with its inertia, allows a gradual increase in temperature and thus facilitates viscosity analysis. To ensure stability, the cylindrical vessel was fastened above the water-filled beaker using a support. The heating plate transfers heat to the water in the beaker. The water, in turn, heats the cylindrical container enclosing the sample. By turning the handle located at the rear of the viscometer, it is possible to lower the rotor, submerging it into the substance whose viscosity is under examination. The distance from the end of the spindle to the bottom of the vessel does not affect the measurement results [25].

The sample was deposited inside the cylindrical container through gentle pressure to avoid undue densification. Under solely gravitational influence, the spindle was slowly lowered into the propellant and submerged up to the middle of the mark engraved on the shaft [26]. After ensuring the stability of the measurement system, a rotor/speed combination must be selected. For each test, a waiting period of 5 min was used before recording the measurements to achieve a stable reading on the screen. The heating plate is set at a constant power, the maximum available one, throughout the test. Upon reaching 90 °C, viscosity measurements were halted. The plate shutdown occurred approximately 10 min later, simulating a potential printing trial, representing the attainment of temperature and the subsequent 10 min required for extrusion. In order to compare the mechanical properties of the heated slurry to the reference values of the more viscous composition at room temperature, mechanical tests were carried out using the slurry heated up to 90 °C.

#### 2.2.2. UV-Curing

The curing of samples for mechanical tests is achieved thanks to a customized illuminating prototype developed by Microdigit (Cazzago San Martino, BS, Italy), where the UV source consists of four individually controllable LED heads in a squared configuration. The LEDs emit a nominal radiant flux of 10 W with a wavelength of 365 nm and standard lenses. The system was encased in a metal box with interlock protection to ensure safety. UV irradiation can be controlled in intensity and time by external signal inputs thanks to a controller that can interface with a computer through a RS232 cable.

The UV curing system was characterized using a Delta Ohm HD2102 radiometer with the LP471UVA probe, as it differs from the one utilized in the previous work [4]. The use of a 3D-printed support for the sensor allows for the collection of data relating to various levels, i.e., distances from the illuminating head. A total of thirty-three points were characterized at various heights, as indicated by a specified matrix. During the post-processing stage, data were analyzed, and a MATLAB script was written to forecast radiation intensity at different levels from the ones that were characterized as well as at points on a single plane. In addition to giving a notion of the actual iso-radiation footprint to attain the most uniform printing area feasible, this also gave information on the intensity values attained at a particular plane and thus the level to select for comparison with the activation energy.

**Figure 5:**
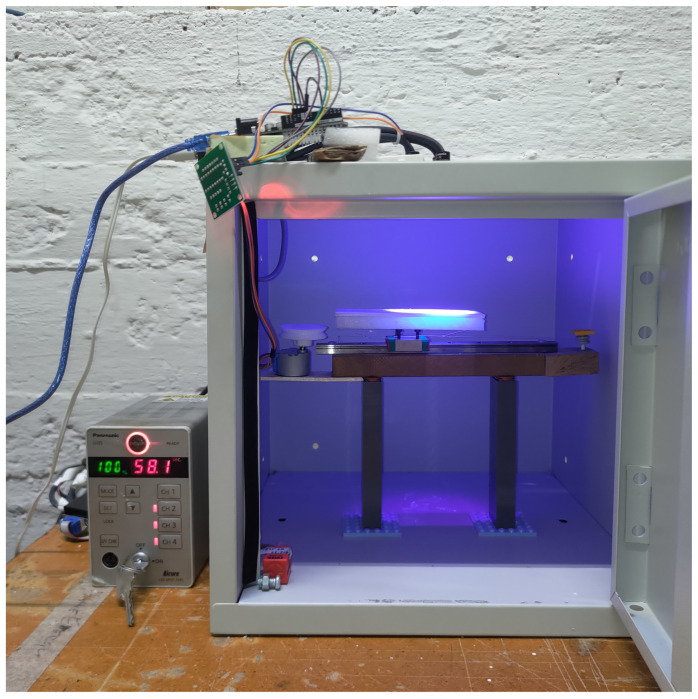
UV curing system with linear guide and adjustable height support.

Having found the optimal point in the x–y plane (the center of the box) and height in the z plane (distance from LED heads equal to 9 cm), the uncured slurry into the mold is cured at 100% intensity for the times specified in Table 2. A linear guide, fixed onto an adjustable height support and controlled through an Arduino board, ensures correct illumination of every point of the specimen. The illuminating prototype and the linear guide are shown in Figure 5.

For multilayer samples used for tensile tests, once the first single layer was exposed to UV light, another mask was stacked on top of the first one in the mold to enable the application of a second layer and further exposure until the required overall thickness was achieved with each layer being cured with a given time as specified in the following. In the previous research phase [4], the production of multilayer samples for tensile tests was carried out using bolted stainless steel molds. To enhance the specimen production rate and optimize the process, filmogenic release agents (polyvinyl alcohol) were tested to facilitate extraction from the metal mold. Eventually, a polymeric mold (TPU material) was employed, which features improved release characteristics and eliminates the need for bolts for fastening, thus enabling faster specimen production, as explained in Section 2.2.3. On the other hand, the samples used for compression tests are monolayer obtained using a TPU mold. The design of the mold, produced using a 3D printer, is extensively explained in Section 2.2.4.

Given the numerous variables and types of tests involved, a test matrix is presented in Table 2 for clarity. Each type of test is characterized by:

- A first letter indicating the type of test (T = tensile, C = compression);
- A second letter indicating the temperature condition (A = ambient temperature, P = printing temperature);
- A progressive numerical code indicating the individual series each with specific characteristics.

Each series is composed of at least three tests. The curing and aging times for the TA series were chosen in accordance with previous work [4], in which it was observed that the surface hardening and the stiffness of the specimens under tensile conditions at ambient temperature showed an asymptotic trend as the curing and aging times both increased. In the present study, the values of 40, 120 and 180 s as the curing time and 24 and 48 h as the aging time were selected to cover a wider range of observation. Hence, the results obtained for the TA series (tensile tests at ambient temperature) as a function of these parameters allowed to investigate the effects of the curing and aging times on the mechanical properties. All the other tests (tensile at printing temperature, compressive at ambient and printing temperatures) were carried out under the identical settings as TA3, which was chosen as a reference since curing and aging conditions were evaluated as optimal, as it will be demonstrated later.

#### 2.2.3. Uniaxial Tensile Tests

Tensile tests were conducted to determine the propellant properties in terms of failure stress and stiffness (Young’s modulus) as well as to investigate the mechanical behavior change due to the heating of the slurry for viscosity improvement. The tests were performed using an electro-mechanical testing machine MTS Insight 1 kN Standard Length equipped with a 100 N load cell.

In line with previous works [4]:

- A test speed of 0.3 mm/s was set;
- The geometry of the multilayer specimen followed the DIN_53504_1994 standard [27];
- In order to have a better clamping to the testing machine and to avoid the specimen breakage in the terminal gripping part, an alternative clamping system based on a metal joint made of an eyelet–carabiner series was set up.

The eyelet was then attached to each side of the specimen using a fast two-component epoxy resin such that a physical and chemical bond with the sample surface was ensured. Next, the metal joint was attached to the fixed and movable part of the machine. The manual machining process was progressively improved to reduce specimen breakage and ensure complete adhesion to the surface and eyelet.

In comparison to our previous work [4], the present project involved a redesign of the sample molds for tensile tests. Specifically, the old method, which relied on a basic metal plate and several screwed-on masks onto which the slide was then placed, proved to be costly in terms of production time and also presented significant problems in the extraction process: one of the encountered challenges was that once the structure was unscrewed, the individual layer sometimes remained attached to one side by the mask but not on the other. The excessive rigidity of the metal structure led to breakage in this situation. The criteria followed for the design of the new mold and, in general, for the production procedure of the test samples, were as follows: ease of use and extraction, integrity of the extracted specimen, number of specimens produced. The new mold, while preserving the old geometry of the samples, was obtained by additive manufacturing using TPU as the material to provide a certain flexibility to facilitate extraction. The procedure involves, once the curing is complete, detaching the individual mask and bending it if necessary. This helps avoid breaks in case there is some adhesion on one side of a mask but not on the other.

**Figure 6:**
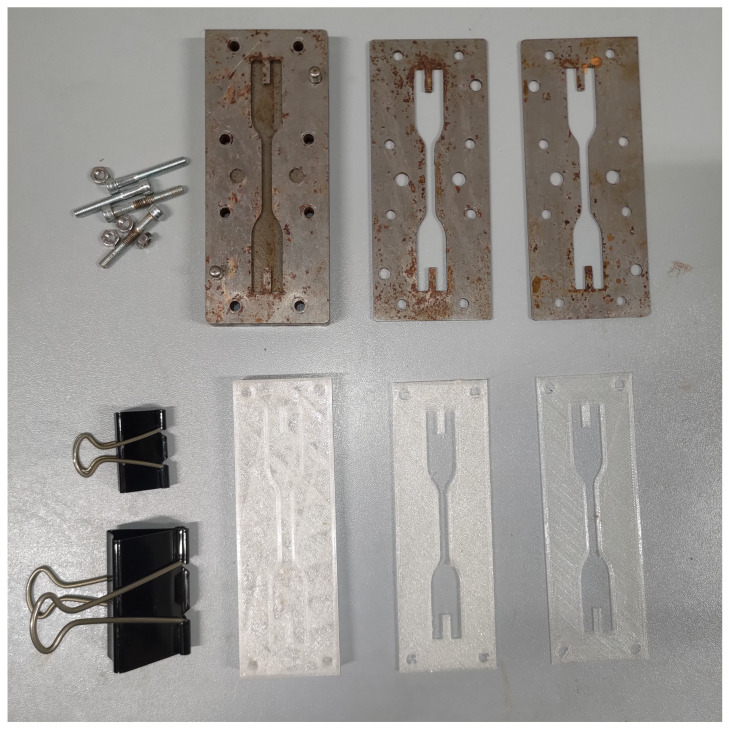
Stainless-steel shaped plates previously used in Ref. [4] (**top**) and TPU-shaped plates used in the present work (**bottom**).

Finally, regarding the adhesion system of the individual masks to the TPU base, screws were abandoned in favor of lateral clamps. This has significantly reduced the production time for a single sample; as in the old configuration, placing the screws took about 2 min each time, and when placing one mask (ply) on top of the other, it was necessary to unscrew and then screw again, significantly extending the times. The comparison between the new mold and the old one can be seen in Figure 6.

The post-processing phase allowed the determination of the applied stress σ due to the knowledge of the applied force *F* provided in output from the machine and specimen section *A* obtained by digital caliper measurements:

(1) $$\sigma =\frac{F}{A}$$

Correspondingly, the value of failure stress was obtained by considering the maximum applied force. The strain ϵ was determined from the displacement ΔL, also provided by the testing machine, in relation to the gauge length $L_{0}$:

(2) $$\epsilon =\frac{\Delta L}{L_{0}}$$

Finally, Young’s modulus was determined by finding the slope of the linear part of the obtained σ-ϵ curves [21].

#### 2.2.4. Compression Tests

Compression tests were carried out using the same testing machine used for tensile cases with a 1000 N load cell.

**Figure 7:**
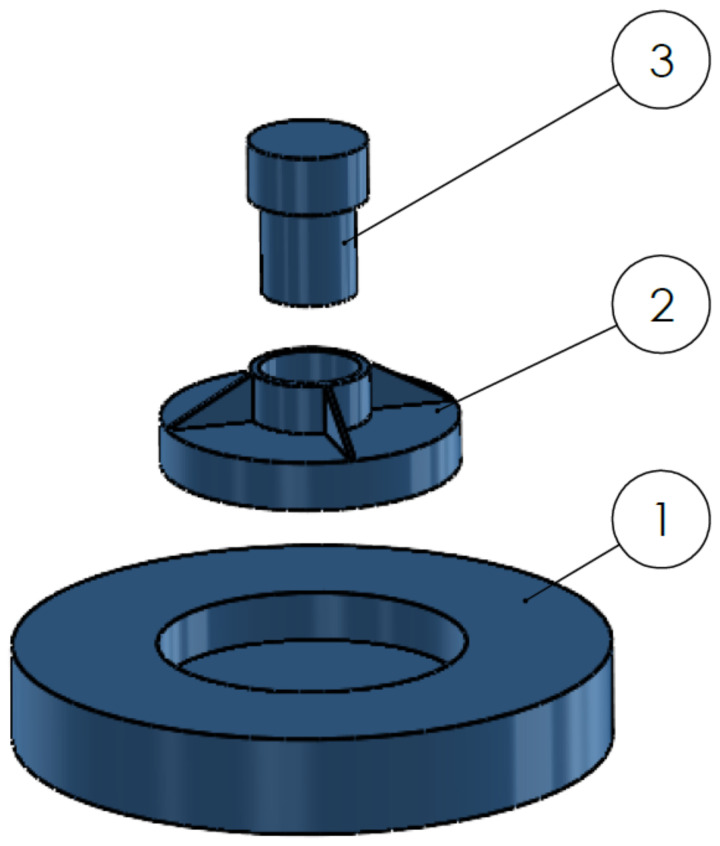
Exploded assembly of the compression mold: (1) bottom plate, (2) actual specimen mold, (3) extraction cylinder.

Regarding the geometry of specimens for compression tests, there is no specific standard available for the current material typology, and several configurations have been found in the literature. In the present work, analysis was conducted on the basis of literature findings and requirements that are specific to the present application. In line with Refs. [28,29], a cylindrical geometry was chosen. The height was set to the same size as the diameter to have fewer possible buckling effects, as reported by Ref. [30]. A diameter of 10 mm was chosen in order to have a good interface with the testing machine and to grant the possibility to create a single layer sample: as also highlighted in [21], the height cannot be larger to ensure complete polymerization by UV light. The mold was designed following the same criteria used for the tensile sample one (ease of use and extraction, integrity of the extracted specimen, number of specimens produced). With reference to Figure 7, the following components can be identified:

1. A circular bottom plate, with a circular recess in the center, to provide stability during the manufacturing.
2. A second component, to be inserted into the circular recess, which contains the actual mold of the specimen (cylinder of diameter and height 10 mm) where the slurry is inserted; it has a through hole, so the lower surface is in direct contact with the surface of the recess in the first component.
3. A cylinder that can be inserted into the through hole, allowing the extraction of the produced sample.

**Figure 8:**
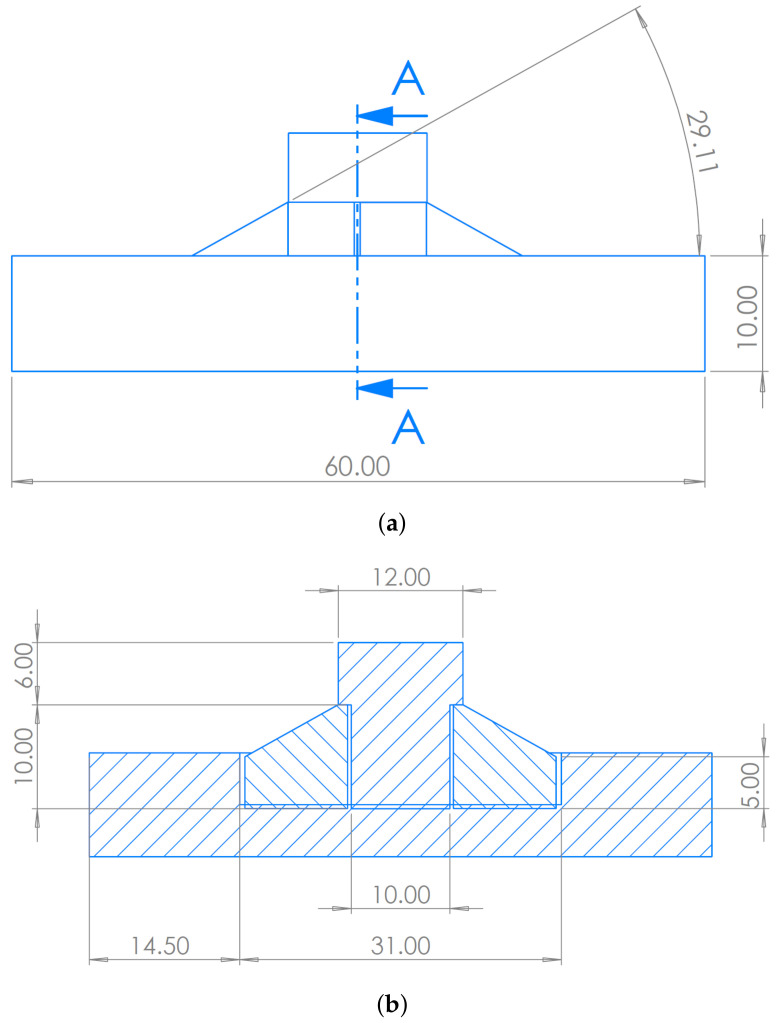
Details of the compression samples mold assembly (unit: mm). (**a**) Side view. (**b**) A-A section.

A detailed view of the assembly is shown in Figure 8.

The mold is produced in TPU through additive manufacturing. The production process involves placing acetate sheets, of negligible size, on the lateral surface of the through hole and on the lower surface of the recess of component 1. These sheets are used to prevent the slurry from sticking to the TPU and causing difficulties in extraction due to the specimen’s modest size. Subsequently, after inserting component 2 into component 1, slurry is deposited into the through hole, and UV rays are used for photo-polymerization. The third component, namely the extraction cylinder, is inserted into one of the two ends of the through hole, allowing for quick and easy extraction. The extracted sample is then freed from the external acetate sheet. The process, resulting from several iterations, has proven effective, especially in terms of the production speed, sample integrity and consistency of the actual dimensions of the produced sample compared to the one designed in CAD.

A preliminary study phase was conducted to determine the testing speed suitable for the current application. In fact, the main effects found on failure stress and stiffness are related to temperature and testing speed as shown in extended articles reported in the literature [28,29,30,31]. Excluding phenomenologies of no interest, such as the absence of descending sections for temperatures far below 0 °C, the following observations can be made:

- At the same operating temperature, an increase in test speed determines higher apparent stiffness;
- At the same test speed, an increase in test temperature determines a lower stiffness.

Analyzing the curves at room temperature [30], it can be seen that the increases in stiffness (slope of the curve) and failure stress have asymptotic behavior as the testing speed increases. Therefore, the value chosen was 5 mm/min to try to obtain intermediate stress and stiffness values with respect to the domain of possible curves. The post-processing phase is the same as the uniaxial tensile tests.

## 3. Results and Discussion

### 3.1. Viscosity Tests

For pure PB measurements, the Arrhenius model has been employed for the experimental assessment of the viscosity curve as a function of temperature. According to this model, viscosity η can be expressed through:

(3) $$ln\left(\eta \right)=ln\left(A_{s}\right)+\frac{E_{A}}{R}\left(\frac{1}{T}\right)$$

where *R*, $E_{a}$ and $A_{s}$ represent, respectively, the gas constant, the Arrhenius activation energy and the pre-exponential entropy factor. The temperature *T* is here expressed in Kelvin degrees. The Arrhenius model applies to resins for which the temperature ranges of validity adhere to the specified condition [32]:

(4) $$T>T_{g}+100\;K$$

Given that PB has a glass transition temperature $T_{g}$ of 203 K (−70 °C) [4], the model closely approximates the behavior beginning at temperatures near 303 K (30 °C).

**Figure 9:**
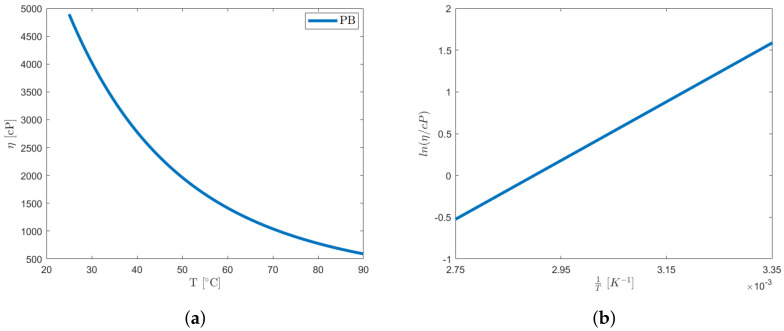
The effect of temperature $\left(T\right)$ on the viscosity $\left(\eta \right)$ of PB. (**a**) Fit of experimental data for pure PB. (**b**) Relation between η and *T* in range of interest.

The plot shown in Figure 9a showcases the viscosity curve as a function of temperature, while Figure 9b displays a standard employed for the graphical assessment of the aforementioned coefficients, which delineate the Arrhenius model [33]. The slope of the straight line is equal to $E_{a}/R$, and the intercept on the ordinate for 1/T=0 represents $ln(A_{s})$. Based on the conducted analyses, an $E_{a}$ value of 29.24 kJ· ${\mathrm{mol}}^{-1}$ and a pre-exponential entropy factor of $ln(A_{s}/\mathrm{cP})=-10.21$ are calculated. These obtained values fall within the valid range of the Arrhenius model [32,33]. The investigation here performed on the binder is of considerable interest for future investigations. The Arrhenius parameters evaluated in this study will be used as a basis to evaluate a rheological equivalent.

The interpolation demonstrates an $R^{2}$ value of 0.9678 and an RMSE of 216.3 cP. The resulting curve aligns coherently with data found in the literature [34,35,36] with viscosity reducing more than eight times with increasing temperature. Since PB is a liquid with a viscosity below 30 Pa·s at high temperatures, effects arising from the utilization of a container with dimensions smaller than the established standard should be taken into account. In the case of the 3.5 cm internal diameter container, this effect remains contained, offsetting the viscosity measure by less than 3% for spindle 3 and by about 1% for spindle 4 [25].

**Figure 10:**
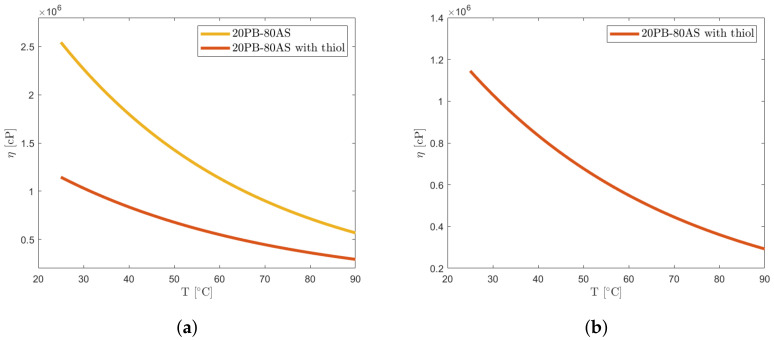
Measurements for the resin with 80% wt solid loading. (**a**) Thiol effect on viscosity of loaded resin. (**b**) PB + AS and thiol viscosity curve.

Expanding the investigation beyond the binder, the test case #2 examined the influence of solid loading on viscosity, focusing on a formulation consisting of 20% PB and 80% AS particles. In order to create a baseline, viscosity measurements of loaded resin were first acquired without the addition of thiol over a temperature range from 25 to 90 °C. This is performed mainly for two reasons: first, to compare the results with the available data in the literature, since current research on the viscosity behavior of loaded resins usually focuses on a combination of binder and powders; secondly, anticipating the effect of thiol on viscosity reduction due to its low viscosity, test case #2 creates a baseline to compare the data from following test case #3. In the second test case, involving the thiol-free formulation, challenges arose as the power control of the heating plate delayed an accurate assessment of viscosity. However, temperature changes during measurements were considered acceptable in the current study phase. A PID controller is now being developed for the study’s subsequent phases with the goal of improving temperature control accuracy for more precise assessments. The loaded thiol-free resin exhibited viscosity values near the upper limit of the instrument’s range ($2\cdot 10^{6}$ cP) even for heated slurry, and the viscosity values at lower temperatures had to be extrapolated. To address these challenges, thiol was introduced, determining the reduction in viscosity and more accurate measurements. The comparison with test case #2 allowed to evaluate thiol’s addition effect. It is important to underline that even if thiols were primarily introduced to allow for photocuring reactions, they have also an important effect on viscosity reduction, acting as a plasticizer. In the future, if needed, the addition of other plasticizers could be investigated to further enhance the printability performance. Figure 10a shows the thiol’s effects on viscosity: at low temperatures, the viscosity of the slurry containing thiols is reduced by half when compared to the slurry without thiols. Moreover, the measured values are in a range where it was possible to have more reliable viscosity data, particularly at lower temperatures. The curve for such a test is displayed in Figure 10b. As can be seen, the measures now fall within the measuring range of the viscometer.

For the composition with thiols, the interpolation is characterized by an $R^{2}$ equal to 0.8781 and an RMSE of 50,947 cP. The wall effect for spindle 4 is negligible in the case of the container with a 3.5 cm internal diameter, and the percentage deviation remains below 1%.

**Figure 11:**
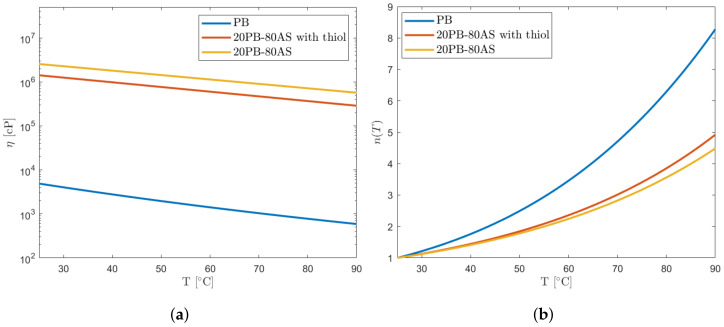
Comparison of viscosity tests and viscosity reduction coefficient *n*. (**a**) Comparison of viscosity–temperature curves. (**b**) Viscosity reduction coefficient *n*.

Finally, it is possible to compare the three curves as displayed in Figure 11a and evaluate the viscosity reduction coefficient *n* as a function of the slurry temperature T (Equation (5)). Note that the unit of measurement for temperatures must be coherent ($T_{amb}=$ 25 °C or $T_{amb}=288.15$ K according to the unit used for *T*).

(5) $$n\left(T\right)=\frac{\eta (T_{amb})}{\eta (T)}$$

The values for the three curves are shown in Table 3. Their comparison is also visualized in Figure 11b.

In the context of our study, the significance of the viscosity curves lies in their critical role in developing the design of a 3D printer prototype specifically designed for the solid propellant formulation here presented. Considering Figure 11b, one notable finding emerges: the addition of thiol has a significant impact on viscosity reduction at higher temperatures, as evidenced by the steepening of the curve. This observation indicates that thiol not only exhibits a plasticizer-like behavior but also amplifies the temperature-induced effects.

### 3.2. Mechanical Testing

#### 3.2.1. Tensile Tests Results

**Figure 12:**
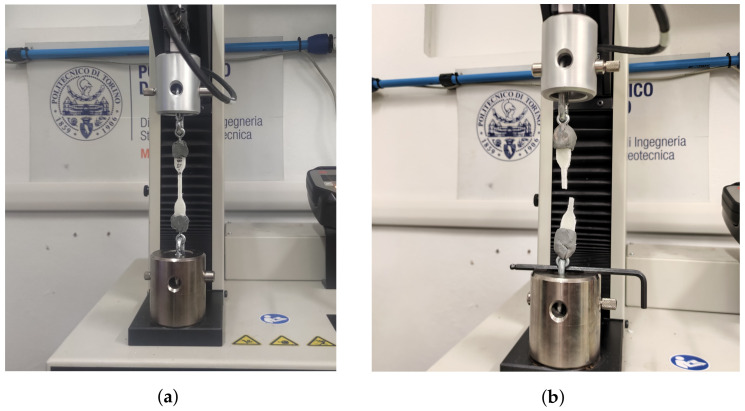
Sample during a tensile test. One can notice the clamping system based on a eyelet-carabiner. (**a**) Sample positioning. (**b**) Sample after test.

A sample during a tensile test is shown in Figure 12a,b. Results from tensile tests performed as described in Section 2.2.3 are shown in Table 4. The TA1 set shows values that do not conform to those found in the literature nor in previous research [4,21]. Indeed, it had already been noted during production that the specimens were brittle with some even flexing under the action of their own weight. For this series, both curing time (40 s) and aging time (24 h) are the lowest in the dataset. Thus, curing time and the effect of aging appear to be important, as also indicated in other work on photo-polymerized solid propellants [8] and classically manufactured composite ones [37,38]. The TA2 set, characterized by a higher curing time and a doubling of aging, shows significantly better results, which are in line with the expected ones. The TA3 set is characterized by the same aging time as TA2, but its curing time is 50% longer. Its values of ultimate stress and Young’s modulus are comparable with TA2, which leads to the conclusion that the effect of aging is preponderant over that of curing times in the UV illuminator for the same mixture. For the same reason, the difference between the results obtained for TA1 and TA2 (due to the increase in both curing and aging times) is similar to that obtained for TA1 and TA3 (due to a further increment in curing time for the same increase in aging as TA2).

**Figure 13:**
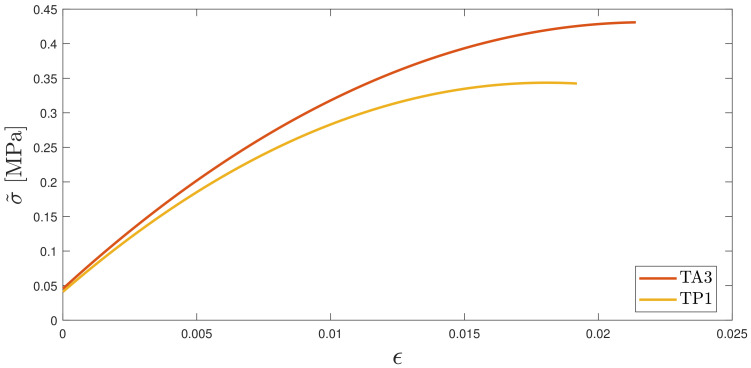
Comparison of fitted tensile stress–strain curves for specimens obtained with unheated slurry (TA3) and heated slurry (TP1). $\tilde{\sigma }$ is the numerical fit of the stress of all the specimens tested in the specified series.

A comparison between TA3 and TP1 (i.e., between specimens produced with slurry at ambient temperature and slurry heated to printing temperature) shows that heating determines a decrease in tensile strength and, to a minor extent, in stiffness. These observed trends are compatible with experimental data obtained at higher ambient temperatures [29,30,31]. A numerical fit $\tilde{\sigma }$ of the obtained curves for each set was performed in order to investigate the overall behavior of the curves slopes in the section of interest (nonlinear and up to break). Results are shown in Figure 13.

#### 3.2.2. Compression Tests Results

**Figure 14:**
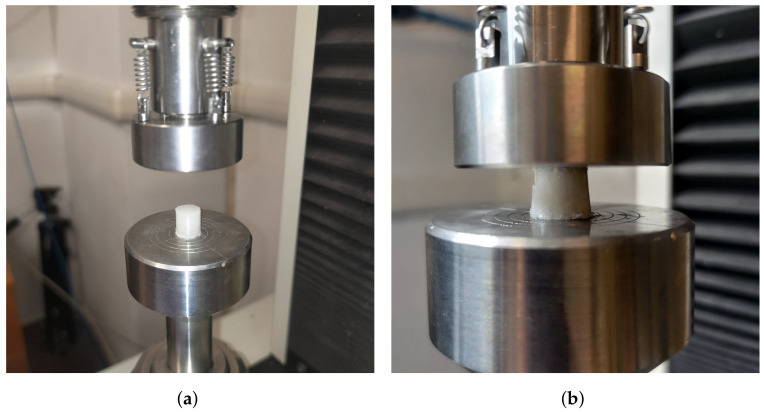
Sample during a compression test. One can notice the typical failure mode of a sample under uniaxial compression. (**a**) Sample positioning. (**b**) Sample after test.

The sample during a compression test is shown in Figure 14a,b. Results are reported in Table 5. 

**Figure 15:**
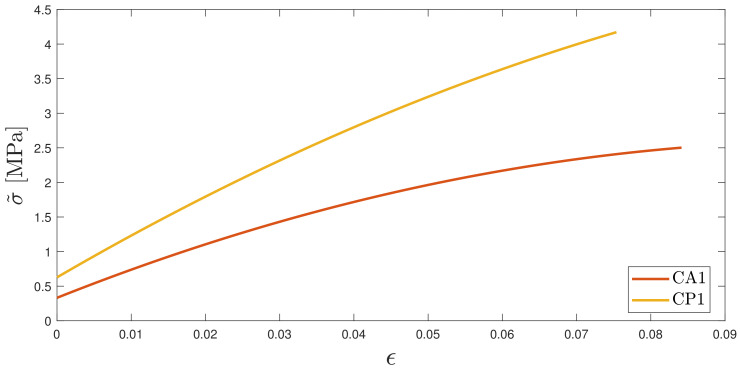
Comparison of fitted compression curves for specimens obtained with unheated slurry (CA1) and heated slurry (CP1). $\tilde{\sigma }$ is the numerical fit of the stress of all the specimens tested in the specified series.

The comparison between CA1 and CP1 in Figure 15 shows that stiffness, ultimate strain and ultimate load increase in the case of slurry heated to printing temperature. Despite a shortage of information in the currently available literature on the influence of pre-heating on compression properties, studies [39,40,41,42] suggest that pre-heating resin composites may result in a more complete polymerization due to a higher degree of conversion, producing a similar effect to that of aging. A numerical fit of the obtained curves $\tilde{\sigma }$ is shown in Figure 15.

## 4. Conclusions

The rheological and mechanical properties of an inert 3D-printable solid propellant are critical when evaluating the printability and performance of solid rocket grains. The study conducted in this work is conceived to properly investigate such properties considering the curing time, aging and temperature effect. Pre-heating was demonstrated to be a feasible method to reduce the viscosity of the uncured propellant. Such an effect was evaluated and compared for pure PB and PB loaded with 80% wt of AS. The viscosity reduction coefficients were computed as a function of temperature increment. The results of viscosity measurements show a viscosity reduction factor larger than 8 for the pure resin when pre-heated from 25 to 90 °C. This value is halved for the complete slurry composition, still guaranteeing a proper rheological behavior for printing applications. Clearly, an increase in temperature of the slurry can influence the process of mixing and curing as well as the resin compatibility with other components in the slurry. Thus, forecasting changes in mechanical properties, tensile and compression tests were performed from specimen obtained from both unheated and heated slurry. First, the curing time and aging effect are investigated. It is found that the post-curing phase, namely the time between production and testing, strongly influences the mechanical properties. Preliminary tests allowed to identify optimal settings (curing time for given radiation flux, aging) for obtaining suitable mechanical properties. The results of mechanical tests show values that align with those of previous research. The comparison performed on mechanical tests of a specimen obtained from the heated slurry show a limited lowering of tensile properties and an increase in compression ones. It may be concluded that pre-heating resin composites may grant a proper viscosity reduction while keeping mechanical properties in the applicability range. The results presented in this work lay the groundwork for the next phase of the project, which will involve developing a 3D printer prototype that combines a UV curing system with a mixing and deposition system.

## Figures and Tables

**Table 1:** Parameters used for viscosity measurements in the test cases for (1) pure resin (PB only), (2) resin with powders (20PB-80AS) and (3) resin with powders and thiol addition. The vessel used for tests has an interal diameter of Φ = 3.5 cm.

Test Case	Formulation	Spindle Type	Rotational Speed (RPM)	Temperature Range (°C)	Test Duration (s)
#1	PB only	LV-3	12–60	2512–90	1200
#2	20PB-80AS	LV-4	0.312–0.6		2000
#3	20PB-80AS with thiol	LV-4	0.6		2100

**Table 2:** Test matrix for mechanical tests.

Code	Series	Curing Time for Each Layer (s)	Aging Time (h)
TA	TA1	40	24
	TA2	120	48
	TA3	180	48
TP	TP1	180	48
CA	CA1	180	48
CP	CP1	180	48

**Table 3:** Viscosity reduction coefficients.

Test Case	Formulation	*n* (90 °C)
#1	PB	8.28
#2	20PB-80AS	4.48
#3	20PB-80AS with thiol	4.92

**Table 4:** Tensile tests results.

Code	Series	Tensile Strength $\sigma_{\mathrm{ymt}}$ (MPa)	Young Modulus $E_{\mathrm{mt}}$ (MPa)	$R^{2}$
TA	TA1	0.136±0.007	3.23	0.983
	TA2	0.386±0.002	22.06	0.982
	TA3	0.431±0.04	19.36	0.843
TP	TP1	0.313±0.01	18.63	0.883

**Table 5:** Compression tests results.

Code	Series	Compression Strength $\sigma_{\mathrm{ymc}}$ (MPa)	Young Modulus $E_{\mathrm{mc}}$ (MPa)	$R^{2}$
CA	CA1	2.29±0.24	28.08	0.866
CP	CP1	3.94±0.24	47.53	0.953

## Data Availability

Data are contained within the article.

## Abbreviations

The following abbreviations are used in this manuscript:

AP	Ammonium Perchlorate
AS	Ammonium Sulfate
BAPO	Bis-(2,4,6-trimethylbenzoyl) phenylphosphine oxide
HTPB	Hydroxyl-Terminated Polybutadiene
PB	Polybutadiene
PPCF	Polypropylene-Reinforced Carbon Fiber
PTTM	Pentaerythritol tetrakis(3-mercaptopropionate)
UV	Ultraviolet
TPU	Thermoplastic Polyurethane
**Nomenclature**
*A*	Area, ${\mathrm{mm}}^{2}$
$A_{s}$	Arrhenius pre-exponential factor, cP
$E_{a}$	Arrhenius activation energy, kJ·${\mathrm{mol}}^{-1}$
*E*	Young’s modulus, MPa
$E_{mc}$	Compression mean Young’s modulus, MPa
$E_{mt}$	Tensile mean Young’s modulus, MPa
*F*	Applied force, N
$L_{0}$	Gauge length, m
ln	Natural logarithm
*R*	Gas constant, J·${\mathrm{mol}}^{-1}\cdot$ $K^{-1}$
$R^{2}$	Coefficient of determination
RMSE	Root mean square error
*T*	Temperature, °C or K
$T_{amb}$	Ambient temperature, °C or K
ΔL	Displacement, m
ϵ	Strain
η	Viscosity, cP
σ	Stress, MPa
$\tilde{\sigma }$	Fitted stress, MPa
$\sigma_{ymc}$	Ultimate mean compression stress, MPa
$\sigma_{ymt}$	Ultimate mean tensile stress, MPa
Φ	Diameter, cm
